# Somatic Mosaic Chromosomal Alterations and Death of Cardiovascular Disease Causes among Cancer Survivors

**DOI:** 10.1158/1055-9965.EPI-22-1290

**Published:** 2023-03-28

**Authors:** Maxine Sun, Marie-Christyne Cyr, Johanna Sandoval, Louis-Philippe Lemieux Perreault, Lambert Busque, Jean-Claude Tardif, Marie-Pierre Dubé

**Affiliations:** 1 Montreal Heart Institute, Montreal, Canada.; 2 Université de Montréal Beaulieu-Saucier Pharmacogenomics Centre, Montreal, Canada.; 3 Department of Medicine, Faculty of Medicine, Université de Montréal, Montreal, Canada.; 4 Hôpital Maisonneuve-Rosemont, Montreal, Canada.

## Abstract

**Background:**

Cancer survivors are at an increased risk of cardiovascular disease (CVD) compared with the general population. We sought to evaluate the impact of mosaic chromosomal alterations (mCA) on death of CVD causes, coronary artery disease (CAD) causes, and of any cause in patients with a cancer diagnosis.

**Methods:**

The study was a prospective cohort analysis of 48,919 UK Biobank participants with a cancer diagnosis. mCAs were characterized using DNA genotyping array intensity data and long-range chromosomal phase inference. Multivariable Cox regression models were used to ascertain the associations of mCAs. Exploratory endpoints included various incident cardiovascular phenotypes.

**Results:**

Overall, 10,070 individuals (20.6%) carried ≥ 1 mCA clone. In adjusted analyses, mCA was associated with an increased risk of death of CAD causes [HR, 1.37; 95% confidence interval (CI), 1.09–1.71; *P* = 0.006]. In sub-analyses, we found that carriers of mCAs diagnosed with kidney cancer had an increased risk of death of CVD causes (HR, 2.03; 95% CI, 1.11–3.72; *P* = 0.022) and CAD causes (HR, 3.57; 95% CI, 1.44–8.84; *P* = 0.006). Women diagnosed with breast cancer who carried a mCA also had a higher risk of death of CAD causes (HR, 2.46; 95% CI, 1.23–4.92; *P* = 0.011).

**Conclusions:**

Among cancer survivors, carriers of any mCA are at an increased risk of CAD death compared with noncarriers. Mechanistic studies should be considered to better ascertain the biological mechanisms underneath the observed associations between mCAs and cardiovascular events for specific cancer types.

**Impact:**

There may be clinical relevance in considering mCAs in patients diagnosed with cancer and undergoing treatment.

## Introduction

The number of survivors of cancer is growing worldwide due to the aging populations and improvements in early cancer detection and treatment modalities (1). It is estimated that over 26 million people in the United States alone will be living with a history of cancer by the year 2040 (2, 3). Among cancer survivors, a pressing clinical problem is their increased predisposition to cardiovascular disease (CVD; refs. 4, 5) and treatment-related cardiac dysfunction (6, 7). Currently, there are no guideline recommendations with respect to CVD screening for patients with cancer, possibly stemming from the lack of CVD-related markers that can better risk-stratify patients with cancer beyond existing cardiovascular risk factors for the general population (8). The identification and development of biomarkers that can eventually be used towards a risk assessment tool for the purpose of discriminating patients diagnosed with cancer who are at a higher risk of CVD may be useful (8).

Clonal hematopoiesis (CH) refers to a population of cells derived from a mutated multipotent stem/progenitor cell occurring in the context of aging (9). CH can be caused by somatic mutation in driver genes (10–14) called clonal hematopoiesis of indeterminate potential (CHIP; ref. 15), or by somatic mosaic chromosomal alterations (mCA; refs. 16–20). Previously, CHIP has been associated with a greater burden of atherosclerotic vessel disease (21), a higher risk of myocardial infarction (22, 23), inflammatory response (24), and death of any cause (11, 12). The presence of CHIP has also been associated with treatment-related adverse outcomes in cancer survivors (25).

Somatic mCAs correspond to large chromosomal gains, loss, or copy neutral losses of heterozygosity which can affect autosomes or sexual chromosomes (bioRxiv 2022.06.24.497515; ref. 26). The most prevalent mCA is the loss of the Y chromosome (mLoY) in aging men (27), which has been associated with all-cause mortality (28, 29), Alzheimer's disease (30), autoimmune disease (31), diabetes (28), and cardiovascular events (32).

Given the links between CH and CVD in the normal aging population, and the paucity of data in cancer survivors, we sought to evaluate the impact of mCAs on the risk of death from CVD causes, from cancer, and of any cause among cancer survivors at risk of CVD. Our hypothesis was that carriers of any mCA would be at higher risk of death from CVD causes compared with their mCA noncarriers.

## Materials and Methods

### Study population

The UK Biobank is a large population-based cohort that includes over 500,000 participants aged between 40 and 70 years old, recruited from 2006 to 2010 (33). Baseline interviews regarding their medical history and environmental exposures were conducted in the UK where blood samples for genotyping were obtained and blood analysis performed. Additional health outcome data, including diagnoses of cancer and CVD, have been linked via UK national registries and hospital records managed by the NHS, and genome-wide genotyping of blood-derived DNA was performed by the UK Biobank using 2 genotyping arrays sharing 95% of marker content (34). The study was approved by the Montreal Heart Institute Research Ethics Committee and complies with the Declaration of Helsinki.

### Determination of mCAs

**Figure 1.**
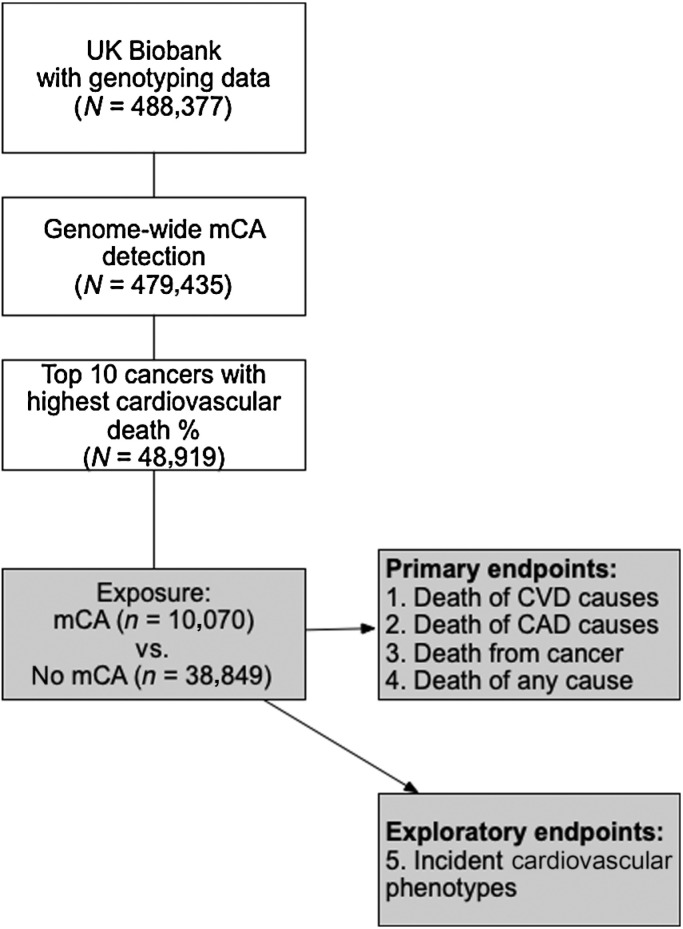
Visual representation of the study flow. Of participants with genotyping and genome-wide mCA detection information available within the UK Biobank, we focused on those with a cancer diagnosis. The exposure of interest (mCA) was assessed for the risks of death of CVD causes, death of CAD causes, death of cancer causes, and death of any cause. The risk of various incident CV phenotypes were considered as exploratory endpoints.

As previously described (19, 20), allele-specific SNP-array intensity data obtained by genotyping blood-derived DNA from UK Biobank participants were used to call mCAs. Specifically, mCAs were determined from genotype intensities log_2_R ratio and B-allele frequency values, which were used to estimate the total and relative allelic intensities, respectively. Re-phasing was conducted using Eagle2 (35) and mCA calling was performed by leveraging long-range phase information searching for allelic imbalances between maternal and paternal allelic fractions across contiguous genomic segments. For the purpose of our study, mCA calls were obtained from dataset Return 3094 from the UK Biobank application 19808 (20, 35). From the genetic data of the UK Biobank, 479,435 individuals who passed the sample quality control criteria, including genotypic-phenotypic sex concordance, and without first- or second-degree relatives in the dataset were considered (Fig. 1). Of those, we identified 48,919 participants with a diagnosis of cancer before or after the baseline assessment visit based on the cancer register, using ICD-9 and ICD-10 diagnostic codes for bladder, larynx, prostate, corpus uteri, rectum, breast, kidneys, non-Hodgkin lymphoma, melanoma of the skin, or lung cancer (Supplementary Table S1). These specific cancer types were selected on the basis of a previous publication which identified the top 10 cancer sites with the largest percentage of deaths attributed to CVD (36).

### Exposure and clinical outcomes

The exposure of interest was the presence of a mCA of any type, which was classified as ≥ 1 or none. mCAs were also categorized as autosomal mCAs, loss of the X chromosome (mLoX), mLoY, and expanded mCAs (defined as mCAs present in at least 10% of peripheral leukocyte DNA indicative of clonal expansion; ref. 37). The study's predetermined primary endpoints (defined in the Supplementary Table S2) consisted of death of CVD causes, death of coronary artery disease (CAD) causes, death from cancer (including recurrent and *de novo* malignancies), and death of any cause based on ICD-10 codes for primary cause of death from the death register records. For each endpoint, the time to death (in years) was calculated from the date of the assessment visit at the time of recruitment into the UK Biobank (baseline) if patients’ cancer diagnosis occurred before baseline, and alternatively, from the date of cancer diagnosis if the cancer diagnosis occurred after baseline. The median follow-up period was 7.2 years. The number of days between a patient's cancer diagnosis date and baseline was recorded, and it was set as 0 if the cancer diagnosis occurred after baseline. For individuals who were not deceased, the end of follow-up was the last date of death registered on the basis of participants’ country of enrolment.

Other exploratory endpoints also considered various *incident* CV phenotypes (38) recorded after patients’ cancer diagnosis date (Supplementary Table S2). For these exploratory endpoints, the time to the event (i.e., incident CV event of interest or death of CVD causes) was calculated from the date of cancer diagnosis. For those who did not experience any incident CV events, censor date was set to the date of death of non-CVD causes, or the last hospitalization date known for each patient.

### Statistical analyses

In our primary analyses focusing on our predetermined primary endpoints, Cox proportional hazards regression models were used to assess the effect of mCA (any vs. none) in the entire cohort of patients with a cancer diagnosis (*n* = 48,919), adjusting for age at baseline, sex, baseline smoking status, treatment with chemotherapy and/or radiotherapy, the number of days between prevalent cancer diagnosis and date of baseline, and principal components for genetic ancestry (PC, 1–10). Sensitivity analyses considered the additional adjustment for baseline measures of alcohol status, use of lipid-lowering medication, type 2 diabetes, hypertension, and body mass index status. The influence of mCA on death of CVD and CAD causes was further tested through competing-risk regression models after adjusting for death of other causes (39).

The association of mCA types with the primary endpoints of interest was also assessed. Exploratory sub-analyses were conducted according to established risk factors of CH, including smoking status, chemotherapy status, and age dichotomized at the 65-year-old cutoff. Corresponding mCA-by-smoking, chemotherapy, and age interaction terms were generated for these analyses, respectively. We further explored the effect of mCA on our primary endpoints according to cancer type. Cox regression models were also used for exploratory analyses of incident CV phenotypes. Finally, to determine whether the potential effects of mCA on outcomes were specific to cancer survivors, a mCA-by-cancer status interaction term was assessed for each of the primary endpoints in the overall population including individuals without any history of cancer. The significance threshold for the primary analyses was revised to account for the multiplicity of endpoints tested using a Bonferroni correction (*P* < 0.05/4 = 0.0125). All other sub-analyses were exploratory in nature and meant to assist in the interpretation of the primary analysis results. All analyses were performed using the survival package in R (version 4.1.2, Bird Hippie). All analytical and summary reports were produced with gtsummary (version 1.6.1; ref. 40).

### Data availability

The data analyzed in this study were obtained from the UK Biobank and are available at https://biobank.ndph.ox.ac.uk/ukb/. For the purpose of our study, mCA calls were obtained from dataset Return 3094 from the UK Biobank application 19808.

## Results

### Baseline characteristics and mCA prevalence

Overall, 48,919 participants were diagnosed with bladder, larynx, corpus uteri, prostate, rectal, breast, kidneys, non-Hodgkin lymphoma, melanoma, or lung cancer within the UK Biobank (Table 1). The presence of ≥ 1 mCA clone was observed in 20.6% (10,070/48,919) of patients (Supplementary Fig. S1A). Of those, 2,432 were autosomal carriers and 1,910 had mCA in ≥ 10% of peripheral leukocytes, defined as an expanded mCA clone. Whereas most carriers had only one mCA, 812 individuals carried ≥ 2 nonoverlapping mCAs. In general, those with mCAs were older (median 64 vs. 61 years), and the prevalence of mCA increased with increasing age categories (Supplementary Fig. S1B). Expectedly, 73.6% of patients with mCA were men, as the majority of mCA carriers was due to mLoY (*n* = 6,558). In comparison, mCA due to mLoX was observed in 1,641 patients (16.3%). Among patients older than 65 years old, the prevalence of mCA according to cancer types was the lowest for breast cancer (14%) and the highest for larynx cancer (43%, Supplementary Fig. S1C).

**Table 1.** Descriptive statistics of patients with a cancer diagnosis stratified according to mCA status.

Characteristic	*N*	Overall, *N* = 48,919^a^	No mCA, *N* = 38,849^a^	Any mCA, *N* = 10,070^a^	*P* ^b^
Age at bsl	48,919				<0.001
*N*		48,919	38,849	10,070	
*N* missing		0	0	0	
Mean (SD)		60 (7)	60 (7)	63 (5)	
Median (IQR)		62 (56, 65)	61 (55, 65)	64 (61, 67)	
Range		40, 71	40, 70	40, 71	
Men	48,919	22,403 (45.8%)	14,989 (38.6%)	7,414 (73.6%)	<0.001
Smoking status	48,624				<0.001
Never smoker		23,865 (49.1%)	19,888 (51.5%)	3,977 (39.7%)	
Previous smoker		19,274 (39.6%)	14,755 (38.2%)	4,519 (45.1%)	
Current smoker		5,485 (11.3%)	3,970 (10.3%)	1,515 (15.1%)	
Unknown		295	236	59	
Bladder cancer	48,919	1,743 (3.6%)	1,191 (3.1%)	552 (5.5%)	<0.001
Larynx cancer	48,919	375 (0.8%)	245 (0.6%)	130 (1.3%)	<0.001
Corpus uteri	48,919	2,331 (4.8%)	2,108 (5.4%)	223 (2.2%)	<0.001
Prostate cancer	48,919	13,274 (27.1%)	8,970 (23.1%)	4,304 (42.7%)	<0.001
Breast cancer	48,919	17,304 (35.4%)	15,678 (40.4%)	1,626 (16.1%)	<0.001
Rectal cancer	48,919	2,268 (4.6%)	1,731 (4.5%)	537 (5.3%)	<0.001
Kidney cancer	48,919	1,910 (3.9%)	1,472 (3.8%)	438 (4.3%)	0.010
Non-Hodgkin lymphoma	48,919	3,038 (6.2%)	2,189 (5.6%)	849 (8.4%)	<0.001
Melanoma	48,919	4,912 (10.0%)	4,031 (10.4%)	881 (8.7%)	<0.001
Lung cancer	48,919	4,215 (8.6%)	3,074 (7.9%)	1,141 (11.3%)	<0.001
Chemotherapy	48,919				<0.001
None		37,512 (76.7%)	29,610 (76.2%)	7,902 (78.5%)	
Yes		11,407 (23.3%)	9,239 (23.8%)	2,168 (21.5%)	
Radiotherapy	48,919				0.036
None		45,846 (93.7%)	36,454 (93.8%)	9,392 (93.3%)	
Yes		3,073 (6.3%)	2,395 (6.2%)	678 (6.7%)	
Prevalent hypertension	48,919	6,077 (12.4%)	4,569 (11.8%)	1,508 (15.0%)	<0.001
Autosomal mCA	48,919				<0.001
Ref.		46,487 (95.0%)	38,849 (100.0%)	7,638 (75.8%)	
Autosomal		2,432 (5.0%)	0 (0.0%)	2,432 (24.2%)	
Loss of X	48,919				<0.001
Ref.		47,278 (96.6%)	38,849 (100.0%)	8,429 (83.7%)	
Loss of X		1,641 (3.4%)	0 (0.0%)	1,641 (16.3%)	
Loss of Y	48,919				<0.001
Ref.		42,361 (86.6%)	38,849 (100.0%)	3,512 (34.9%)	
Loss of Y		6,558 (13.4%)	0 (0.0%)	6,558 (65.1%)	
Expanded mCA	48,919				<0.001
Ref.		47,009 (96.1%)	38,849 (100.0%)	8,160 (81.0%)	
Expanded mCA		1,910 (3.9%)	0 (0.0%)	1,910 (19.0%)	
Death of CVD causes	48,919	813 (1.7%)	550 (1.4%)	263 (2.6%)	<0.001
Death of CAD causes	48,919	368 (0.8%)	226 (0.6%)	142 (1.4%)	<0.001
Death from cancer	48,919	8,433 (17.2%)	6,254 (16.1%)	2,179 (21.6%)	<0.001
Death of any cause	48,919	10,632 (21.7%)	7,785 (20.0%)	2,847 (28.3%)	<0.001

Abbreviations: bsl., baseline; IQR, interquartile range; Ref., referent category.

^a^ *n* (%).

^b^Wilcoxon rank sum test; Pearson *χ*^2^ test.

### mCA and the risk of death

**Figure 2.**
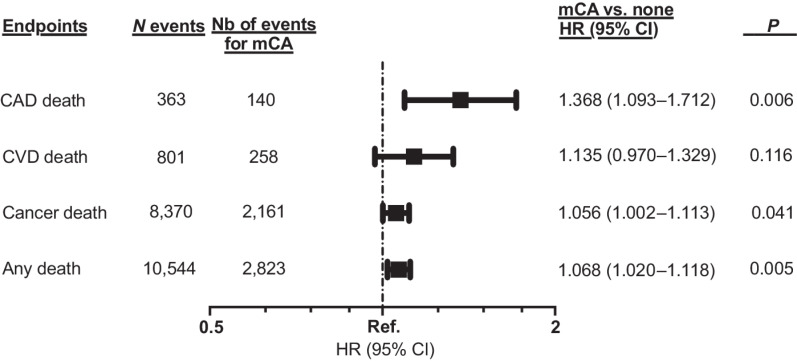
Multivariable Cox regression models evaluating the effect of mCAs on death of CVD causes, of CAD causes, from cancer, and death of any cause. All models are adjusted for age at baseline, sex, smoking status, chemotherapy, radiotherapy, number of days between the date of recruitment and the date of cancer diagnosis, and principal components 1 to 10. Ref., referent category (1.0).

In adjusted Cox regression analyses, mCA was associated with an increased risk of death of CAD causes [HR, 1.37; 95% confidence interval (CI), 1.09–1.71; *P* = 0.006; Fig. 2] and death of any cause (HR, 1.07; 95% CI, 1.02–1.12; *P* = 0.005; Fig. 2). These effects were maintained in sensitivity analyses after the additional adjustment for baseline measures of alcohol status, use of lipid-lowering medication, type 2 diabetes, hypertension, and body mass index (Supplementary Table S3). Multivariable competing-risk regression analyses replicated the effect of mCA on the risk of death of CAD causes, despite adjustment for death of non-CAD causes and other covariates (HR_competing-risk_, 1.33; 95% CI, 1.06–1.68; *P* = 0.014; Supplementary Table S4). The effect of mCA on death of CVD causes (*P*_interaction_ = 0.044) and of any cause (*P*_interaction_ = 0.011) modulated according to smoking status (Supplementary Table S5). In contrast, the impact of mCA was not influenced by use of chemotherapy for any of our primary endpoints (all *P*_interaction_ ≥ 0.05; Supplementary Table S6).

Different types of mCAs had different effects on the study endpoints. Notably, autosomal mCAs were associated with death of CVD causes, with death from cancer, as well as with death of any cause (Supplementary Table S7). mCA due to mLoX was associated with death of CAD causes (Supplementary Table S8), and mCA due to mLoY was associated with death of any cause (Supplementary Table S9). Expanded mCAs taken individually had no statistically significant impact on the primary endpoints (Supplementary Table S10). When considering the study population of 479,435 participants with and without a history of any cancer, the interaction terms between cancer and mCA were significant for the endpoints of death from cancer and death of any cause (Supplementary Table S11).

### mCA and the risk of death according to cancer site

In multivariable analyses where the effect of mCA was assessed in subgroups according to cancer type, we found that carriers of mCAs diagnosed with kidney cancer had an increased risk of death of CVD causes (HR, 2.03; 95% CI, 1.11–3.72; *P* = 0.022) and CAD causes (HR, 3.57; 95% CI, 1.44–8.84; *P* = 0.006; Supplementary Tables S12 and S13) compared with their mCA noncarrier counterparts diagnosed with kidney cancer. Women diagnosed with breast cancer who carried a mCA also had a higher risk of death of CAD causes (HR, 2.46; 95% CI, 1.23–4.92; *P* = 0.011). mCA had no significant impact on deaths from cancer in either cancer type (Supplementary Table S14). Women carriers of mCA diagnosed with corpus uteri cancer had an increased risk of death of any cause (HR, 1.35; 95% CI, 1.01–1.80; *P* = 0.042; Supplementary Table S15).

### mCA and the risk of incident CV endpoints (exploratory analyses)

**Figure 3.**
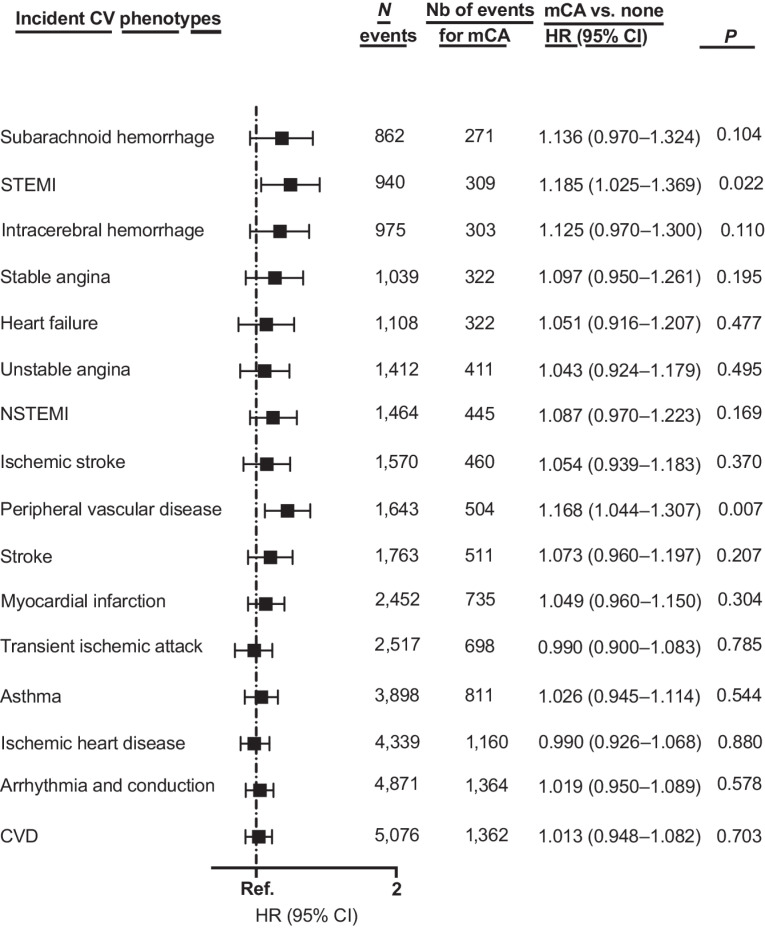
Multivariable Cox regression models evaluating the effect of mCAs on incident cardiovascular phenotypes. All models are adjusted for age at baseline, sex, smoking status, chemotherapy, radiotherapy, number of days between the date of recruitment and the date of cancer diagnosis, and principal components 1 to 10. MI: myocardial infarction, NSTEMI, non-ST-elevation myocardial infarction; STEMI, ST-elevation myocardial infarction; TIA, transient ischemic attack.

In the overall cohort of cancer survivors (*n* = 48,919), carriers of mCA had a higher risk of incident ST-elevation myocardial infarction (STEMI; HR, 1.19; 95% CI, 1.03–1.37; *P* = 0.022) and peripheral vascular disease (HR, 1.17; 95% CI, 1.04–1.31; *P* = 0.007; Fig. 3) than mCA noncarriers. Similar findings were obtained when the analyses were restricted to those aged ≥ 65 years old (Supplementary Table S16).

## Discussion

Here, we assessed the impact of somatic mCAs, a subtype of CH, on the risks of death of CVD causes, CAD causes, from cancer, and death of any cause focusing exclusively on individuals diagnosed with one of 10 cancer types known to have the highest reported rates of CVD deaths (36). Primarily, we found that carriers of mCAs had a notable increased risk of death of CAD causes in all patients diagnosed with cancer. The increased risk of death of CAD causes for carriers of mCAs persisted even after adjusting for death of other causes as a competing event, which included cancer-related deaths. While mCAs were also associated with death of any causes overall, a finding likely driven by death from cancer-related causes, its impact was more subdued. Furthermore, sub-analyses did not reveal a significant association between mCA and cancer-specific deaths for any of the cancer types. On the other hand, mCAs portended to higher risks of both death of CVD and CAD causes in patients diagnosed with kidney cancer and higher risks of death of CAD causes for women diagnosed with breast cancer. Our findings also revealed that some of the associations were more impactful according to mCA subtypes. For example, autosomal mCAs were associated with the risk of death of CVD causes, whereas mCA due to the mLoX was associated with the risk of death of CAD causes, and mCA due to the mLoY was associated with the risk of death of any causes.

In addition, exploratory analyses suggest that carriers of mCAs were at higher risk of incident STEMI and peripheral vascular disease; and for cancer survivors aged > 65 years, at a higher risk of incident NSTEMI, stable angina, intracerebral hemorrhage, and subarachnoid hemorrhage. In comparison, a previous mCA study focusing on myocardial infarction and stroke did not find any significant associations when focusing on carriers of expanded mCAs, regardless of cancer status (20).

In comparison with previous studies that focused on the general population and found that mCAs were not associated with CAD (19, 37), the current study suggests that any mCA carrier status may have prognostic ability of other CV phenotypes in cancer survivors. Past research that found a relationship between CH and CAD were focused on CHIP mutations. One such study correlated mutations in *DNMT3A* and *TET2* with chronic heart failure in patients with STEMI (*n* = 485; ref. 41). In another study which included approximately 50,000 samples with exome sequencing data, Zekavat and colleagues (bioRxiv 2022.06.24.497515) found that carriers of CHIP mutations had higher risks of peripheral artery disease.

Second, when considering cancer survivors, it is possible that cancer-directed treatments may have perpetuated the influence of mCA on outcomes (25). However, our sub-analyses which included a chemotherapy-by-mCA interaction term failed to reach statistical significance, albeit an observed near twofold increased risk of death of CAD causes for mCA carriers. Previous work has supported the hypothesis that chemotherapy may lead to clonal expansion of mutations in apoptosis or DNA repair gens (*TP53*, *PPM1D*; refs. 25, 42), and that CHIP mutations caused by these genes could worsen the risk of atherosclerosis (bioRxiv 2022.06.24.497515). It is possible that chemotherapy usage may not be well captured in the current database. Furthermore, without knowledge on tumor aggressiveness in the current database, it was not possible to exclude the presence of a selection bias where carriers of mCAs may be at a more advanced stage of the disease or have a more aggressive tumor histology, which may make them more likely to undergo additional lines of chemotherapy/radiotherapy, which could have contributed to their risks of CVD. Because other studies that examined the influence of chemotherapy on CH were focused on CHIP mutations, it will be critical to evaluate whether cancer-directed treatments have any additive or synergistic influence on mCAs. Further research will be needed to understand the impact of mCAs on CVD outcomes following modern standards of care, such as treatment with immune checkpoint inhibitors. In that scenario, mCA carriers may benefit from an in-depth preventive cardiology approach prior to treatment where multidisciplinary cardio-oncology care could mitigate such risks.

Third, we found that the effect of mCA on the risks of death of CVD and CAD causes, as well as of death of any cause was specific to previous smokers. This upholds the findings of a recent study which established strong causal associations between smoking and mCAs, and postulated that smoking may contribute to the selection of clones bearing somatic mutations (43). Indeed, we found that cancer survivors who are carriers of mCAs may face added risks if they smoked in the past. Additional work is required to better evaluate how smoking habits can impact mCAs and the downstream consequences of such mutations, especially in patients diagnosed with cancer.

The generalizability of our findings may be limited as the influence of mCA was not consistent across all examined cancer types. This was not entirely unexpected given the heterogeneous biology of cancers included and distinct mechanisms of mCAs with respect to these cancers and treatments that patients received. Nonetheless, the associations observed within this study may inform future studies focusing on the effect of mCA and the risks of death from CVD causes for specific cancer types.

Our study has other limitations. First, our study did not consider CHIP mutations. While the association of CHIP mutations with CVD has been established, it has not been formally tested in patients diagnosed exclusively with non-hematologic cancer. There is reason to believe that carriers of CHIP mutations in addition to being diagnosed with cancer may face higher risks of CVD. In addition, the synergistic effect of both mCA and CHIP mutation carriers among cancer survivors will be of interest to explore in future studies. Second, it has been established that detectable mLoY and mLoX chromosomes increase with age, with more age-related pathologies in older individuals than in younger individuals (44, 45). Unexpectedly, the association between the mLoY and death of CVD causes was not significant in the current analysis that focused on cancer survivors. The nonsignificant association may have been related to the mLoY definition we used, where a larger percentage threshold of blood cells lacking chromosome Y may be more relevant. For example, a recent analysis found that regardless of their cancer status, men with mLoY chromosome in 40% or more of leukocytes displayed a 31% increased risk of dying from any disease related to the circulatory system based on survival data from the UK Biobank (45). In contrast, the relative risk per 1% increase of detectable mLoY and the risk of death from CVD causes was more subdued (HR, 1.0054; *P* = 0.001; ref. 45). We did also note that mLoY chromosome was rather impactful for cancer-specific mortality, which may be relevant in considering the significant association found between mLoY and death of any cause. Third, mCAs were detected using allele-specific SNP microarray intensity data obtained by genotyping blood-derived DNA samples from participants, as previously described (19, 20), allowing more mCA events than previously developed methodologies (16, 17, 46). Alternatively, the use of whole-genome or whole-exome sequencing to detect mosaicism have also been proposed. However, such approaches can be limited in their ability to detect mCAs at low cell fractions. Also, mCA status was evaluated only at study entry. Reasonably, clones carrying a mCA may undergo rapid changes following various environmental exposures (e.g., smoking, cancer-directed therapy). Under this premise, serial measurements of mCA may better help inform the dose–response effect of such factors and mCA. In addition, while adjustment was made for chemotherapy and radiotherapy treatment, some individuals may have undergone such treatments in the outpatient setting, which would not have been properly captured using inpatient procedural codes. In that regard, we also did not have information on the number of cancer-directed treatment cycles, or dosage of therapies in these patients. Furthermore, with regard to variable adjustment, other lifestyle and environmental factors may have also been considered. Future studies focusing specifically on how modifiable lifestyle behaviors (e.g., sleep, diet, physical activity) or the use of medication (e.g., anti-hypertensives, lipid-lowering) may impact the development of CH can have considerable clinical relevance to cancer survivors. Finally, although the current study is the first to assess the relationship between mCAs and CVD in cancer survivors, we did not have available independent cohorts to conduct an external replication of the observed associations.

In conclusion, our study showed that mCAs are associated with higher risks of death due to CAD causes among cancer survivors. Future studies should focus on specific cancer types and their treatments to better ascertain the effect of mCAs on the risk of CVD, and to evaluate whether they constitute a useful biomarker in the management of cancer survivors.

## Supplementary Material

Supplementary Table 2 Supplementary Table 2: ICD-9 and ICD-10 diagnostic and procedure codes for inpatient cardiovascular-related endpoints and for the primary endpoints

Supplementary Table 3 Supplementary Table 3: Sensitivity analyses by Cox regression considering additional covariates

Supplementary Table 4 Supplementary Table 4: Multivariable competing-risks regression model for the prediction of death of CVD causes and CAD causes

Supplementary Table 5 Supplementary Table 5: Cox regression analyses evaluating the effect of mosaic chromosomal alterations on the risk of death of cardiovascular disease causes, coronary artery disease causes, from cancer, and any cause of death stratified by smoking status groups

Supplementary Table 6 Supplementary Table 6: Cox regression analyses evaluating the effect of mosaic chromosomal alterations on the risk of death of cardiovascular disease causes, coronary artery disease causes, from cancer, and any cause of death by chemotherapy status

Supplementary Table 7 Supplementary Table 7: The effect of autosomal mosaic chromosomal alterations on death of cardiovascular disease causes, coronary artery disease causes, from cancer and any cause of death

Supplementary Table 8 Supplementary Table 8: The effect of mosaic loss of X chromosome on death of cardiovascular disease causes, coronary artery disease causes, from cancer, and any cause of death

Supplementary Table 9 Supplementary Table 9: The effect of mosaic loss of Y chromosome on death of cardiovascular disease causes, coronary artery disease causes, from cancer and any cause of death

Supplementary Table 10 Supplementary Table 10: The effect of expanded mosaic chromosomal alterations on death of cardiovascular disease causes, coronary artery disease causes, from cancer, and any cause of death

Supplementary Table 11 Supplementary Table 11: The effect of mosaic chromosomal alterations (mCA) on death of cardiovascular disease causes, coronary artery disease causes, from cancer, and any-cause death with a mCA-by-cancer status interaction term in all patients

Supplementary Table 12 Supplementary Table 12: Cox regression analyses of the effect of mosaic chromosomal alterations on the risk of death of cardiovascular disease causes per cancer type

Supplementary Table 13 Supplementary Table 13: Cox regression analyses for the effect of mosaic chromosomal alterations on the risk of death of coronary artery disease causes by cancer type

Supplementary Table 14 Supplementary Table 14: Cox regression analyses of the effect of mosaic chromosomal alterations on the risk of death from cancer according to cancer type

Supplementary Table 15 Supplementary Table 15: Cox regression analyses evaluating the effect of mosaic chromosomal alterations on the risk of any cause of death by cancer type

Supplementary Table 16 Supplementary Table 16: Cox regression analyses for the effect of mosaic chromosomal alterations on the risk of incident cardiovascular endpoints aged ≥65 years old (n=15,273)

Supplementary Figure 1 Supplementary Figure 1: Visual depiction of the proportion of patients with at least 1 mosaic chromosomal alteration

Supplementary Figure 2 Supplementary Figure 2: Visual mapping of mosaic chromosomal alterations

Supplementary Table 1 Supplementary Table 1: Primary diagnostic codes for selected cancers
