# Supplementary material for: Somatic Mosaic Chromosomal Alterations and Death of Cardiovascular Disease Causes among Cancer Survivors
Source: Cancer Epidemiol Biomarkers Prev. 2023 Mar 28;32(6):776–83. doi: 10.1158/1055-9965.EPI-22-1290 (PMC10233351; doi:10.1158/1055-9965.EPI-22-1290)
Supplement: Supplementary Table 2 — ICD-9 and ICD-10 diagnostic and procedure codes for inpatient cardiovascular-related endpoints and for the primary endpoints [file epi-22-1290_supplementary_table_2_suppst2.docx]

**Supplementary Table 2.** ICD-9 and ICD-10 diagnostic and procedure codes for inpatient cardiovascular-related endpoints^39^ and for the primary endpoints

| **Description** | **ICD-9** | **ICD-10** |
| --- | --- | --- |
| Death for cardiovascular  disease causes |  | I00_I99 |
| Death for coronary  artery disease causes |  | I20, I21, I22, I23, I24 or I25 |
| Death from cancer |  | C01x, C02x, C03x, C04x, C05x, C06x, C07x, C08x, C09x, C10x, C11x, C12, C13x, C14x, C15x, C16x, C17x, C18x, C19, C20, C21x, C22x, C23x, C25x, C26x, C30x, C31x, C32x, C33, C34x, C37, C38x, C39x, C40x, C41x, C43x, C45x, C46x, C47x, C48x, C49x, C50x, C51x, C52, C53x, C54x, C55, C56, C57x, C48, C60x, C61, C62x, C64, C65, C66, C67x, C68x, C69x, C70x, C71x, C73, C74x, C75x, C76x, C78x, C79x, C80x, C81x, C82x, C83x, C85x, C85x, C86x, C88x, C90x, C91x, C92x, C93x, C94x, C95x, C96x, C97x, D45, D473, D752, D474, D758, D471, D479 |
| Cardiovascular disease  (including angina pectoris,  STEMI, NSTEMI, other  acute ischemic heart  diseases, chronic ischemic  heart disease, cardiac arrest, nontraumatic subarachnoid  hemorrhage, nontraumatic intracerebral hemorrhage, cerebral infarction) | 410.x, 411.x, 412.x, 413.x, 414.x, 429.79, 430.x, 431.x, 432.x, 433.x, 434.x, 435.x, 436.x, 437.x, 438.x | I20.x, I21.x, I22.x, I23.x, I24.1, I25.x, I46, I60.x, I61.x, I63.x |
| Ischemic heart disease | 410.x - 414.x | I20.x - I25.x |
| Myocardial infarction | 410.x, 411.x, 412.x, 429.79 | I21.x, I22.x, I23.x, I24.1, I25.2 |
| STEMI | 410.0 - 410.6, 410.8 - 410.9 | I21.0 - I21.3, I22.0, I22.1, I22.8 |
| NSTEMI | 410.7 | I21.4, I21.9, I22.9 |
| Stable angina | 413.1, 413.9 | I20.1, I20.8, I20.9 |
| Unstable angina | 411.1, 411.81, 411.89 | I20.0, I24.0, I24.8, I24.9 |
| Stroke | 430.x, 431.x, 432.x, 433.x, 434.x, 435.x, 436.x, 437.x, 438.x | I60.x, I61.x, I63.x, I64.x |
| Ischemic stroke | 434.x,436.x | I63.x, I64.x |
| Intracerebral hemorrhage | 431.x | I61.x |
| Subarachnoid hemorrhage | 430.x | I60.x |
| Transient ischemic attack | 435.x | G45.0, G45.1, G45.2, G45.8, G45.9 |
| Heart Failure | 428.x, 425.x | I50.x, I42.0 |
| Peripheral vascular disease | 250.6x, 440.2x, 443.1, 443.8, 443.9 | I73.x, I74.3, I74.4, I74.5 |
| Arrhythmia and conduction disorders (incl. atrial fibrillation) | 426.x, 427.x | I44.x, I48.x |
| Asthma | 493.x | J45.x, J46.x |
|  |  |  |
