# Supplementary material for: Somatic Mosaic Chromosomal Alterations and Death of Cardiovascular Disease Causes among Cancer Survivors
Source: Cancer Epidemiol Biomarkers Prev. 2023 Mar 28;32(6):776–83. doi: 10.1158/1055-9965.EPI-22-1290 (PMC10233351; doi:10.1158/1055-9965.EPI-22-1290)
Supplement: Supplementary Table 3 — Sensitivity analyses by Cox regression considering additional covariates [file epi-22-1290_supplementary_table_3_suppst3.docx]

| **Supplementary Table 3.** Sensitivity analyses by Cox regression considering additional covariates | | | | | | | |
| --- | --- | --- | --- | --- | --- | --- | --- |
|  |  |  |  |  |  |  |  |
| **Characteristic** | **N** | **Event N** | **HR***^1^* | **95% CI***^1^* | **p-value** |  |  |
| **Time to CVD death** | | | | | |  |  |
| **mCA** |  |  |  |  |  |  |  |
| No mCA | 38,612 | 543 | — | — |  |  |  |
| Any mCA | 10,011 | 258 | 1.154 | 0.985, 1.351 | 0.076 |  |  |
| **Time to CAD death** | | | | | |  |  |
| **mCA** |  |  |  |  |  |  |  |
| No mCA | 38,612 | 223 | — | — |  |  |  |
| Any mCA | 10,011 | 140 | 1.379 | 1.102, 1.726 | 0.005 |  |  |
| **Time to cancer death** | | | | | |  |  |
| **mCA** |  |  |  |  |  |  |  |
| No mCA | 38,612 | 6209 | — | — |  |  |  |
| Any mCA | 10,011 | 2161 | 1.069 | 1.015, 1.127 | 0.012 |  |  |
| **Time to any death** | | | | | |  |  |
| **mCA** |  |  |  |  |  |  |  |
| No mCA | 38,612 | 7721 | — | — |  |  |  |
| Any mCA | 10,011 | 2823 | 1.081 | 1.032, 1.132 | <0.001 |  |  |

*Models adjusted for age at baseline, sex, smoking status, alcohol status, chemotherapy, radiotherapy, prevalent hypertension, prevalent high cholesterol, prevalent use of lipid-lowering medication, body mass index, number of days between date of recruitment and date of cancer diagnosis, and genotyping principal components 1-10. ^1^ CAD: coronary artery disease, CI: confidence interval, CVD: cardiovascular disease, HR: hazard ratio, mCA: mosaic chromosomal alterations*
