# Supplementary material for: Somatic Mosaic Chromosomal Alterations and Death of Cardiovascular Disease Causes among Cancer Survivors
Source: Cancer Epidemiol Biomarkers Prev. 2023 Mar 28;32(6):776–83. doi: 10.1158/1055-9965.EPI-22-1290 (PMC10233351; doi:10.1158/1055-9965.EPI-22-1290)
Supplement: Supplementary Table 4 — Multivariable competing-risks regression model for the prediction of death of CVD causes and CAD causes [file epi-22-1290_supplementary_table_4_suppst4.docx]

| **Supplementary Table 4.** Multivariable competing-risks regression model for the prediction of death of | | | | | | | |  |
| --- | --- | --- | --- | --- | --- | --- | --- | --- |
| CVD causes and CAD causes | | |  |  |  |  |  |  |
| **Cardiovascular disease (CVD)** |  |  |  |  |  |  |  |  |
|  | **HR** | **95% CI** | ***P*** |  |  |  |  |  |
| **mCA vs. none** | 1.109 | 0.946, 1.300 | 0.200 |  |  |  |  |  |
|  |  |  |  |  |  |  |  |  |
| **Coronary artery disease (CAD)** |  |  |  |  |  |  |  |  |
|  | **HR** | **95% CI** | ***P*** |  |  |  |  |  |
| **mCA vs. none** | 1.333 | 1.060, 1.677 | 0.014 |  |  |  |  |  |

All models adjusted for age at baseline, sex, chemotherapy, days elapsed between prevalent cancer diagnosis date and study entry date, smoking status, receipt of chemotherapy, principal components 1 thru 10, and death from non-CVD or CAD causes. HR: hazard ratio, CI: confidence interval
