# Supplementary material for: Somatic Mosaic Chromosomal Alterations and Death of Cardiovascular Disease Causes among Cancer Survivors
Source: Cancer Epidemiol Biomarkers Prev. 2023 Mar 28;32(6):776–83. doi: 10.1158/1055-9965.EPI-22-1290 (PMC10233351; doi:10.1158/1055-9965.EPI-22-1290)
Supplement: Supplementary Table 6 — Cox regression analyses evaluating the effect of mosaic chromosomal alterations on the risk of death of cardiovascular disease causes, coronary artery disease causes, from cancer, and any cause of death by chemotherapy status [file epi-22-1290_supplementary_table_6_suppst6.docx]

**Supplementary Table 6.** Cox regression analyses evaluating the effect of mosaic chromosomal alterations on the risk of death of cardiovascular disease causes, coronary artery disease causes, from cancer, and any cause of death by chemotherapy status.

| **Characteristic** | Time to CVD death | | | | | Time to CAD death | | | | | Time to cancer death | | | | | Time to any death | | | | |
| --- | --- | --- | --- | --- | --- | --- | --- | --- | --- | --- | --- | --- | --- | --- | --- | --- | --- | --- | --- | --- |
|  |  | **Event N** | **HR***^1^* | **95% CI***^1^* | **p-value** |  | **Event N** | **HR***^1^* | **95% CI***^1^* | **p-value** |  | **Event N** | **HR***^1^* | **95% CI***^1^* | **p-value** |  | **Event N** | **HR***^1^* | **95% CI***^1^* | **p-value** |
| **Never treated with chemotherapy** |  |  |  |  |  |  |  |  |  |  |  |  |  |  |  |  |  |  |  |  |
| No mCA (n=29,425) |  | 437 | — | — |  |  | 188 | — | — |  |  | 3080 | — | — |  |  | 4260 | — | — |  |
| Any mCA (n=7,853) |  | 203 | 1.082 | 0.907, 1.291 | 0.384 |  | 109 | 1.242 | 0.970, 1.593 | 0.088 |  | 1170 | 1.069 | 0.990, 1.148 | 0.071 |  | 1682 | 1.065 | 1.002, 1.131 | 0.042 |
| **Previously treated with chemotherapy** |  |  |  |  |  |  |  |  |  |  |  |  |  |  |  |  |  |  |  |  |
| No mCA (n=9,188) |  | 106 | — | — |  |  | 35 | — | — |  |  | 3129 | — | — |  |  | 3461 | — | — |  |
| Any mCA (n=2,158) |  | 55 | 1.381 | 0.970, 1.964 | 0.073 |  | 31 | 2.151 | 1.270, 3.641 | 0.004 |  | 991 | 1.033 | 0.960, 1.115 | 0.415 |  | 1141 | 1.058 | 0.980, 1.137 | 0.128 |

Models adjusted for age at baseline, sex, radiotherapy, number of days between date of cancer diagnosis and date of baseline, genotyping principal components 1 thru 10. *^1^ CAD: coronary artery disease, CI: confidence interval, CVD: cardiovascular disease, HR: hazard ratio, mCA: mosaic chromosomal alterations*
