# Supplementary material for: Somatic Mosaic Chromosomal Alterations and Death of Cardiovascular Disease Causes among Cancer Survivors
Source: Cancer Epidemiol Biomarkers Prev. 2023 Mar 28;32(6):776–83. doi: 10.1158/1055-9965.EPI-22-1290 (PMC10233351; doi:10.1158/1055-9965.EPI-22-1290)
Supplement: Supplementary Table 7 — The effect of autosomal mosaic chromosomal alterations on death of cardiovascular disease causes, coronary artery disease causes, from cancer and any cause of death [file epi-22-1290_supplementary_table_7_suppst7.docx]

**Supplementary Table 7.** The effect of autosomal mosaic chromosomal alterations on death of cardiovascular disease causes, coronary artery disease causes, from cancer and any cause of death.

| **Characteristic** | **N** | **Event N** | **HR***^1^* | **95% CI***^1^* | **p-value** |
| --- | --- | --- | --- | --- | --- |
| **Time to CVD death** | | | | | |
| **Autosomal mCA** |  |  |  |  |  |
| Ref. | 46,203 | 739 | — | — |  |
| Autosomal | 2,421 | 62 | 1.353 | 1.043, 1.754 | 0.023 |
| **Time to CAD death** | | | | | |
| **Autosomal mCA** |  |  |  |  |  |
| Ref. | 46,203 | 333 | — | — |  |
| Autosomal | 2,421 | 30 | 1.444 | 0.990, 2.100 | 0.055 |
| **Time to cancer death** | | | | | |
| **Autosomal mCA** |  |  |  |  |  |
| Ref. | 46,203 | 7841 | — | — |  |
| Autosomal | 2,421 | 529 | 1.137 | 1.041, 1.242 | 0.004 |
| **Time to any death** | | | | | |
| **Autosomal mCA** |  |  |  |  |  |
| Ref. | 46,203 | 9870 | — | — |  |
| Autosomal | 2,421 | 674 | 1.143 | 1.057, 1.236 | <0.001 |

Models adjusted for age at baseline, sex, smoking status, chemotherapy, radiotherapy, number of days between date of cancer diagnosis and date of study recruitment, genotyping principal components 1 thru 10. *^1^ CAD: coronary artery disease, CI: confidence interval, CVD: cardiovascular disease, HR: hazard ratio, mCA: mosaic chromosomal alterations, Ref.: referent category includes no mCA or mCAs that were not autosomal*
