# Supplementary material for: Somatic Mosaic Chromosomal Alterations and Death of Cardiovascular Disease Causes among Cancer Survivors
Source: Cancer Epidemiol Biomarkers Prev. 2023 Mar 28;32(6):776–83. doi: 10.1158/1055-9965.EPI-22-1290 (PMC10233351; doi:10.1158/1055-9965.EPI-22-1290)
Supplement: Supplementary Table 8 — The effect of mosaic loss of X chromosome on death of cardiovascular disease causes, coronary artery disease causes, from cancer, and any cause of death [file epi-22-1290_supplementary_table_8_suppst8.docx]

**Supplementary Table 8.** The effect of mosaic loss of X chromosome on death of cardiovascular disease causes, coronary artery disease causes, from cancer, and any cause of death.

| **Characteristic** | **N** | **Event N** | **HR***^1^* | **95% CI***^1^* | **p-value** |  |
| --- | --- | --- | --- | --- | --- | --- |
| **Time to CVD death** | | | | | |  |
| **chrX** |  |  |  |  |  |  |
| Ref. | 24,745 | 258 | — | — |  |  |
| Loss of X | 1,632 | 27 | 1.194 | 0.801, 1.780 | 0.384 |  |
| **Time to CAD death** | | | | | |  |
| **chrX** |  |  |  |  |  |  |
| Ref. | 24,745 | 78 | — | — |  |  |
| Loss of X | 1,632 | 15 | 2.022 | 1.157, 3.535 | 0.013 |  |
| **Time to cancer death** | | | | | |  |
| **chrX** |  |  |  |  |  |  |
| Ref. | 24,745 | 3715 | — | — |  |  |
| Loss of X | 1,632 | 277 | 1.005 | 0.889, 1.137 | 0.932 |  |
| **Time to any death** | | | | | |  |
| **chrX** |  |  |  |  |  |  |
| Ref. | 24,745 | 4497 | — | — |  |  |
| Loss of X | 1,632 | 343 | 1.000 | 0.896, 1.118 | 0.994 |  |

*Models adjusted for age at baseline, smoking status, chemotherapy, radiotherapy, number of days between date of recruitment and date of cancer diagnosis, and genotyping principal components 1-10. ^1^ CAD: coronary artery disease, CI: confidence interval, CVD: cardiovascular disease, HR: hazard ratio, mCA: mosaic chromosomal alterations, Ref.: referent category includes no mCA or mCAs that were not loss of X chromosome*
