# Supplementary material for: Somatic Mosaic Chromosomal Alterations and Death of Cardiovascular Disease Causes among Cancer Survivors
Source: Cancer Epidemiol Biomarkers Prev. 2023 Mar 28;32(6):776–83. doi: 10.1158/1055-9965.EPI-22-1290 (PMC10233351; doi:10.1158/1055-9965.EPI-22-1290)
Supplement: Supplementary Table 9 — The effect of mosaic loss of Y chromosome on death of cardiovascular disease causes, coronary artery disease causes, from cancer and any cause of death [file epi-22-1290_supplementary_table_9_suppst9.docx]

**Supplementary Table 9.** The effect of mosaic loss of Y chromosome on death of cardiovascular disease causes, coronary artery disease causes, from cancer and any cause of death.

| **Characteristic** | **N** | **Event N** | **HR***^1^* | **95% CI***^1^* | **p-value** |
| --- | --- | --- | --- | --- | --- |
| **Time to CVD death** | | | | | |
| **chrY** |  |  |  |  |  |
| Ref. | 15,733 | 333 | — | — |  |
| Loss of Y | 6,514 | 183 | 1.03 | 0.856, 1.238 | 0.757 |
| **Time to CAD death** | | | | | |
| **chrY** |  |  |  |  |  |
| Ref. | 15,733 | 170 | — | — |  |
| Loss of Y | 6,514 | 100 | 1.147 | 0.890, 1.478 | 0.289 |
| **Time to cancer death** | | | | | |
| **chrY** |  |  |  |  |  |
| Ref. | 15,733 | 2882 | — | — |  |
| Loss of Y | 6,514 | 1496 | 1.065 | 0.998, 1.136 | 0.056 |
| **Time to any death** | | | | | |
| **chrY** |  |  |  |  |  |
| Ref. | 15,733 | 3717 | — | — |  |
| Loss of Y | 6,514 | 1987 | 1.077 | 1.019, 1.140 | 0.009 |

*Models adjusted for age at baseline, smoking status, chemotherapy, radiotherapy, number of days between date of recruitment and date of cancer diagnosis, and genotyping principal components 1-10.* HR = Hazard Ratio, CI = Confidence Interval, *CAD: coronary artery disease, CI: confidence interval, CVD: cardiovascular disease, HR: hazard ratio, mCA: mosaic chromosomal alterations, Ref.: referent category includes no mCA or mCAs that were not loss of Y chromosome*
