# Supplementary material for: Somatic Mosaic Chromosomal Alterations and Death of Cardiovascular Disease Causes among Cancer Survivors
Source: Cancer Epidemiol Biomarkers Prev. 2023 Mar 28;32(6):776–83. doi: 10.1158/1055-9965.EPI-22-1290 (PMC10233351; doi:10.1158/1055-9965.EPI-22-1290)
Supplement: Supplementary Table 10 — The effect of expanded mosaic chromosomal alterations on death of cardiovascular disease causes, coronary artery disease causes, from cancer, and any cause of death [file epi-22-1290_supplementary_table_10_suppst10.docx]

**Supplementary Table 10.** The effect of expanded mosaic chromosomal alterations on death of cardiovascular disease causes, coronary artery disease causes, from cancer, and any cause of death.

| **Characteristic** | **N** | **Event N** | **HR***^1^* | **95% CI***^1^* | **p-value** |  |
| --- | --- | --- | --- | --- | --- | --- |
| **Time to CVD death** | | | | | |  |
| **Expanded mCA** |  |  |  |  |  |  |
| Ref. | 46,722 | 768 | — | — |  |  |
| Expanded mCA | 1,902 | 33 | 1.170 | 0.819, 1.672 | 0.388 |  |
| **Time to CAD death** | | | | | |  |
| **Expanded mCA** |  |  |  |  |  |  |
| Ref. | 46,722 | 347 | — | — |  |  |
| Expanded mCA | 1,902 | 16 | 1.624 | 0.97, 2.724 | 0.066 |  |
| **Time to cancer death** | | | | | |  |
| **Expanded mCA** |  |  |  |  |  |  |
| Ref. | 46,722 | 8035 | — | — |  |  |
| Expanded mCA | 1,902 | 335 | 0.997 | 0.892, 1.114 | 0.958 |  |
| **Time to any death** | | | | | |  |
| **Expanded mCA** |  |  |  |  |  |  |
| Ref. | 46,722 | 10115 | — | — |  |  |
| Expanded mCA | 1,902 | 429 | 1.025 | 0.929, 1.131 | 0.626 |  |

*Models adjusted for age at baseline, sex, smoking status, chemotherapy, radiotherapy, number of days between date of recruitment and date of cancer diagnosis, and genotyping principal components 1-10.* HR = Hazard Ratio, CI = Confidence Interval, *CAD: coronary artery disease, CI: confidence interval, CVD: cardiovascular disease, HR: hazard ratio, mCA: mosaic chromosomal alterations, Ref.: referent category includes no mCA or not expanded mCAs*
