# Supplementary material for: Somatic Mosaic Chromosomal Alterations and Death of Cardiovascular Disease Causes among Cancer Survivors
Source: Cancer Epidemiol Biomarkers Prev. 2023 Mar 28;32(6):776–83. doi: 10.1158/1055-9965.EPI-22-1290 (PMC10233351; doi:10.1158/1055-9965.EPI-22-1290)
Supplement: Supplementary Table 11 — The effect of mosaic chromosomal alterations (mCA) on death of cardiovascular disease causes, coronary artery disease causes, from cancer, and any-cause death with a mCA-by-cancer status interaction term in all patients [file epi-22-1290_supplementary_table_11_suppst11.docx]

**Supplementary Table 11.** The effect of mosaic chromosomal alterations (mCA) on death of cardiovascular disease causes, coronary artery disease causes, from cancer, and any-cause death with a mCA-by-cancer status interaction term in all patients

| **Characteristic** | **N** | **Event N** | **HR***^1^* | **95% CI***^1^* | **p-value** |
| --- | --- | --- | --- | --- | --- |
| **Time to CVD death** | | | | | |
| **Cancer status * mCA** | 479,435 | 7315 | 1.016 | 0.896, 1.154 | 0.800 |
| **Time to CAD death** | | | | | |
| **Cancer status * mCA** | 479,435 | 3793 | 0.995 | 0.831, 1.193 | 0.961 |
| **Time to any cancer death** | | | | | |
| **Cancer status * mCA** | 479,435 | 17680 | 0.62 | 0.541, 0.711 | <0.001 |
| **Time to any death** | | | | | |
| **Cancer status * mCA** | 479,435 | 35527 | 0.718 | 0.685, 0.754 | <0.001 |

*Models adjusted for age at baseline, sex, smoking status, mCA, any cancer, principal components 1-10, and a mCA-by-cancer status interaction term. Of note, any cancer here includes all cancer diagnoses identified within the cancer register. CAD: coronary artery disease, CI: confidence interval, CVD: cardiovascular disease, HR: hazard ratio, mCA: mosaic chromosomal alterations*
