# Supplementary material for: Somatic Mosaic Chromosomal Alterations and Death of Cardiovascular Disease Causes among Cancer Survivors
Source: Cancer Epidemiol Biomarkers Prev. 2023 Mar 28;32(6):776–83. doi: 10.1158/1055-9965.EPI-22-1290 (PMC10233351; doi:10.1158/1055-9965.EPI-22-1290)
Supplement: Supplementary Table 14 — Cox regression analyses of the effect of mosaic chromosomal alterations on the risk of death from cancer according to cancer type [file epi-22-1290_supplementary_table_14_suppst14.docx]

**Supplementary Table 14.** Cox regression analyses of the effect of mosaic chromosomal alterations on the risk of death from cancer according to cancer type.

| **Characteristic** | **N** | **Event N** | **HR***^1^* | **95% CI***^1^* | **p-value** |
| --- | --- | --- | --- | --- | --- |
| **Bladder cancer** | | | | | |
| mCA |  |  |  |  |  |
| No mCA | 1,184 | 319 | — | — |  |
| Any mCA | 550 | 169 | 1.079 | 0.887, 1.313 | 0.445 |
| **Larynx cancer** | | | | | |
| mCA |  |  |  |  |  |
| No mCA | 243 | 57 | — | — |  |
| Any mCA | 129 | 23 | 0.684 | 0.407, 1.149 | 0.151 |
| **Corpus uteri** | | | | | |
| mCA |  |  |  |  |  |
| No mCA | 2,092 | 291 | — | — |  |
| Any mCA | 221 | 45 | 1.353 | 0.980, 1.859 | 0.063 |
| **Prostate cancer** | | | | | |
| mCA |  |  |  |  |  |
| No mCA | 8,906 | 958 | — | — |  |
| Any mCA | 4,277 | 571 | 1.025 | 0.922, 1.140 | 0.644 |
| **Rectal cancer** | | | | | |
| mCA |  |  |  |  |  |
| No mCA | 1,723 | 378 | — | — |  |
| Any mCA | 535 | 144 | 1.168 | 0.950, 1.435 | 0.138 |
| **Breast cancer** | | | | | |
| mCA |  |  |  |  |  |
| No mCA | 15,606 | 1680 | — | — |  |
| Any mCA | 1,618 | 189 | 1.059 | 0.909, 1.234 | 0.464 |
| **Kidney cancer** | | | | | |
| mCA |  |  |  |  |  |
| No mCA | 1,463 | 368 | — | — |  |
| Any mCA | 437 | 137 | 0.99 | 0.805, 1.218 | 0.923 |
| **Non-Hodgkin lymphoma** | | | | | |
| mCA |  |  |  |  |  |
| No mCA | 2,173 | 419 | — | — |  |
| Any mCA | 845 | 227 | 1.15 | 0.970, 1.362 | 0.106 |
| **Melanoma** | | | | | |
| mCA |  |  |  |  |  |
| No mCA | 4,018 | 325 | — | — |  |
| Any mCA | 876 | 109 | 0.99 | 0.778, 1.249 | 0.907 |
| **Lung cancer** | | | | | |
| mCA |  |  |  |  |  |
| No mCA | 3,039 | 2000 | — | — |  |
| Any mCA | 1,129 | 778 | 0.98 | 0.894, 1.071 | 0.643 |

Models adjusted for age at baseline, sex (except for breast and prostate cancer), smoking status, chemotherapy, radiotherapy, number of days elapsed between date of recruitment and date of cancer diagnosis, and genotyping principal components 1 thru 10. HR: hazard ratio, CI: confidence interval, mCA: mosaic chromosomal alterations
