# Supplementary material for: Somatic Mosaic Chromosomal Alterations and Death of Cardiovascular Disease Causes among Cancer Survivors
Source: Cancer Epidemiol Biomarkers Prev. 2023 Mar 28;32(6):776–83. doi: 10.1158/1055-9965.EPI-22-1290 (PMC10233351; doi:10.1158/1055-9965.EPI-22-1290)
Supplement: Supplementary Table 16 — Cox regression analyses for the effect of mosaic chromosomal alterations on the risk of incident cardiovascular endpoints aged ≥65 years old (n=15,273) [file epi-22-1290_supplementary_table_16_suppst16.docx]

**Supplementary Table 16.** Cox regression analyses for the effect of mosaic chromosomal alterations on the risk of incident cardiovascular endpoints aged ≥65 years old (n=15,273).

| **Characteristic** | **N** | **Event N** | **HR***^1^* | **95% CI***^1^* | **p-value** |
| --- | --- | --- | --- | --- | --- |
| **CVD** | | | | | |
| mCA | 15,273 | 2365 | 1.043 | 0.950, 1.141 | 0.356 |
| **Ischemic heart disease** | | | | | |
| mCA | 15,273 | 2032 | 1.034 | 0.939, 1.139 | 0.498 |
| **MI** | | | | | |
| mCA | 15,273 | 1230 | 1.114 | 0.990, 1.259 | 0.081 |
| **STEMI** | | | | | |
| mCA | 15,273 | 487 | 1.229 | 1.015, 1.488 | 0.034 |
| **NSTEMI** | | | | | |
| mCA | 15,273 | 757 | 1.203 | 1.031, 1.404 | 0.019 |
| **Stable angina** | | | | | |
| mCA | 15,273 | 538 | 1.204 | 1.003, 1.445 | 0.046 |
| **Unstable angina** | | | | | |
| mCA | 15,273 | 741 | 1.124 | 0.960, 1.316 | 0.145 |
| **Stroke** | | | | | |
| mCA | 15,273 | 908 | 1.074 | 0.931, 1.240 | 0.328 |
| **Ischemic stroke** | | | | | |
| mCA | 15,273 | 829 | 1.085 | 0.934, 1.260 | 0.287 |
| **Intracerebral hemorrhage** | | | | | |
| mCA | 15,273 | 506 | 1.223 | 1.013, 1.477 | 0.036 |
| **Subarachnoid hemorrhage** | | | | | |
| mCA | 15,273 | 462 | 1.234 | 1.013, 1.503 | 0.037 |
| **Heart failure** | | | | | |
| mCA | 15,273 | 581 | 1.121 | 0.938, 1.340 | 0.209 |
| **TIA** | | | | | |
| mCA | 15,273 | 1317 | 1.033 | 0.916, 1.164 | 0.599 |
| **Peripheral vascular disease** | | | | | |
| mCA | 15,273 | 838 | 1.197 | 1.033, 1.388 | 0.017 |
| **Arrhythmia and conduction** | | | | | |
| mCA | 15,273 | 2420 | 1.033 | 0.945, 1.128 | 0.475 |
| **Asthma** | | | | | |
| mCA | 15,273 | 1426 | 1.004 | 0.890, 1.132 | 0.953 |

Models adjusted for age at baseline, sex, smoking status, chemotherapy, radiotherapy, number of days between cancer diagnosis and date of study recruitment, and genotyping principal components 1 thru 10. HR: hazard ratio, CI: confidence interval, mCA: mosaic chromosomal alterations, MI: myocardial infarction, STEMI: ST-elevation myocardial infarction, NSTEMI: non-ST-elevation myocardial infarction, CVD: cardiovascular disease, TIA: transient ischemic attack
