# Supplementary material for: Somatic Mosaic Chromosomal Alterations and Death of Cardiovascular Disease Causes among Cancer Survivors
Source: Cancer Epidemiol Biomarkers Prev. 2023 Mar 28;32(6):776–83. doi: 10.1158/1055-9965.EPI-22-1290 (PMC10233351; doi:10.1158/1055-9965.EPI-22-1290)
Supplement: Supplementary Figure 1 — Visual depiction of the proportion of patients with at least 1 mosaic chromosomal alteration [file epi-22-1290_supplementary_figure_1_suppsf1.docx]

**Supplementary Figure 1.** Visual depiction of the proportion of patients with at least 1 mosaic chromosomal alteration in the overall cohort (A), of the proportion of patients with at least one mosaic chromosomal alteration stratified according to age groups (B), and of the proportion of patients with at least one mosaic chromosomal alteration stratified according to cancer types (C).


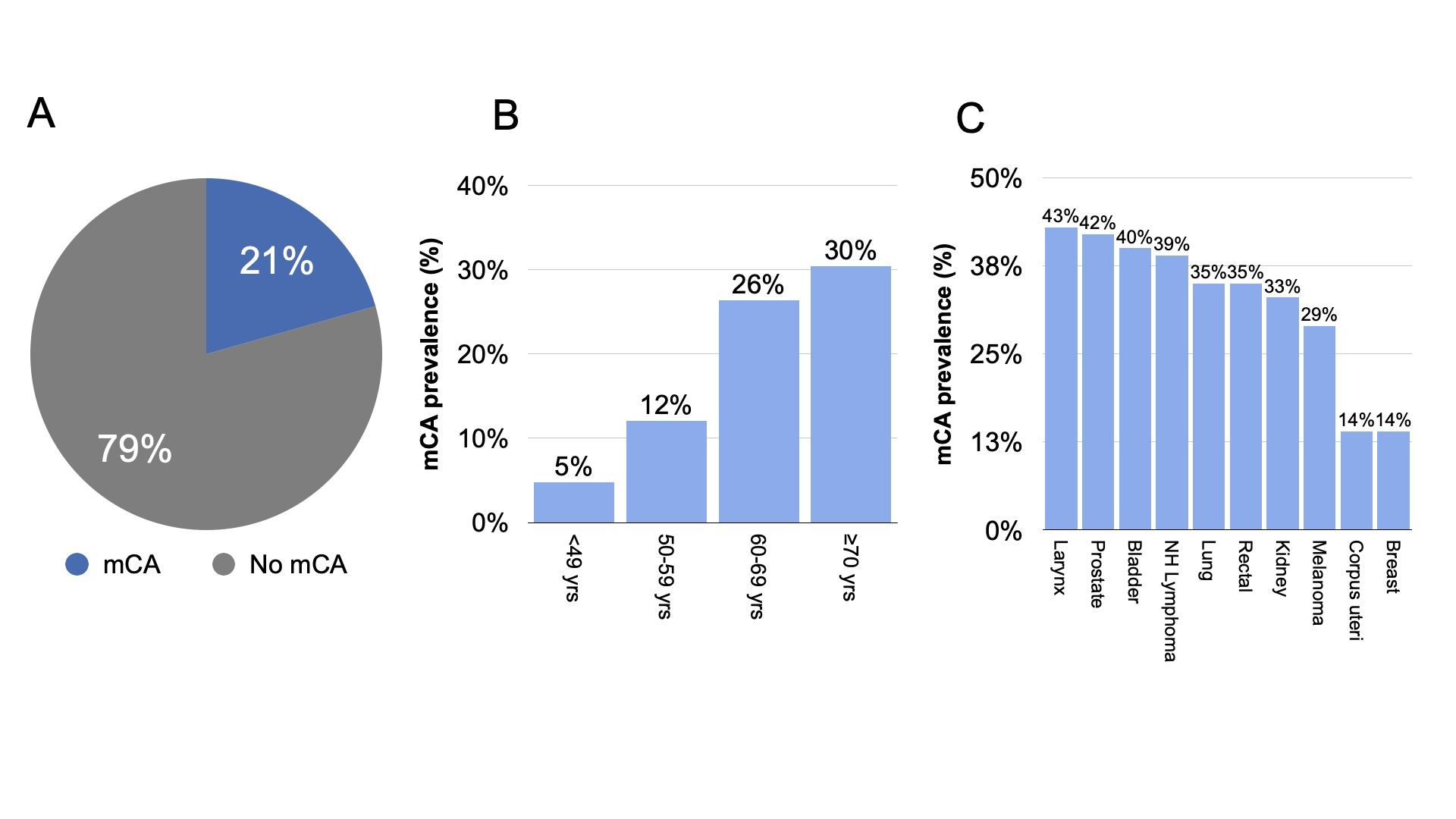
mCA: mosaic chromosomal alteration.
