# Supplementary material for: Somatic Mosaic Chromosomal Alterations and Death of Cardiovascular Disease Causes among Cancer Survivors
Source: Cancer Epidemiol Biomarkers Prev. 2023 Mar 28;32(6):776–83. doi: 10.1158/1055-9965.EPI-22-1290 (PMC10233351; doi:10.1158/1055-9965.EPI-22-1290)
Supplement: Supplementary Figure 2 — Visual mapping of mosaic chromosomal alterations [file epi-22-1290_supplementary_figure_2_suppsf2.docx]

**Supplementary Figure 2.** Visual mapping of mosaic chromosomal alterations (mCAs) detected across the genome among individuals who died of coronary artery disease (CAD) causes.


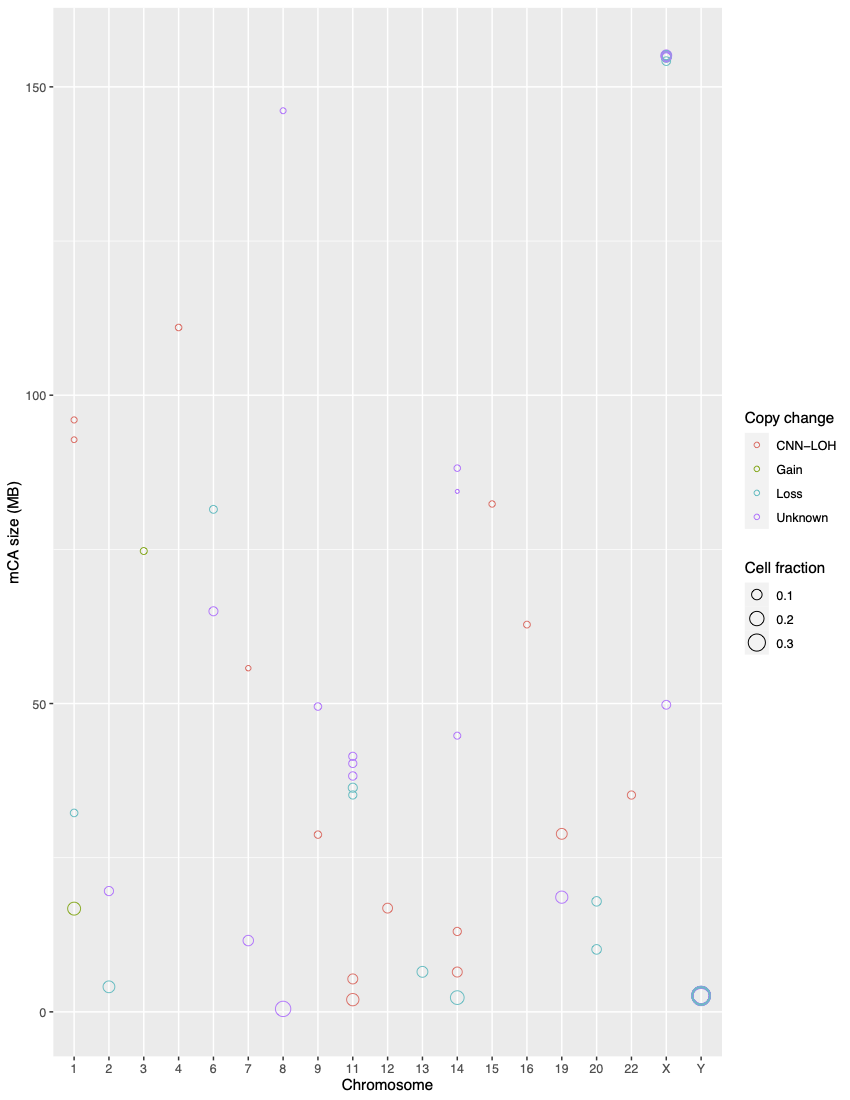


A total of 155 mCA events, including 5 and 102 events on the X and Y chromosome, respectively, were detected across 140 unique participants. Each point represents one mCA event, with the x-axis as the chromosome, y-axis as the mCA size in mega-bases of DNA (MB), color coded by copy number (loss, gain, copy-number neutral loss of heterozygosity [CNN-LOH], unknown), and the size of the point as the cell fraction of that mCA.
