# Supplementary material for: Somatic Mosaic Chromosomal Alterations and Death of Cardiovascular Disease Causes among Cancer Survivors
Source: Cancer Epidemiol Biomarkers Prev. 2023 Mar 28;32(6):776–83. doi: 10.1158/1055-9965.EPI-22-1290 (PMC10233351; doi:10.1158/1055-9965.EPI-22-1290)
Supplement: Supplementary Table 1 — Primary diagnostic codes for selected cancers [file epi-22-1290_supplementary_table_1_suppst1.docx]

| **Supplementary Table 1.** Primary diagnostic codes for selected cancers^37^ | | | |  |  |  |  |  |
| --- | --- | --- | --- | --- | --- | --- | --- | --- |
|  | **ICD-9** | **ICD-10** |  |  |  |  |  |  |
| Urinary bladder | 188x | C67x |  |  |  |  |  |  |
| Larynx | 161.0, 161.1, 161.3, 161.9 | C32x |  |  |  |  |  |  |
| Prostate | 185 | C61 |  |  |  |  |  |  |
| Corpus uteri | 182 | C54x |  |  |  |  |  |  |
| Rectal | 154.1 | C20 |  |  |  |  |  |  |
| Breast | 174x | C50x |  |  |  |  |  |  |
| Kidney | 189 | C64 |  |  |  |  |  |  |
| Non-Hodgkin Lymphoma | 200.0 to 200.7, 202.0, 202.1, 202.2, 202.7 | C82x, C83x, C84x, C85x |  |  |  |  |  |  |
| Melanoma of the skin | 172.x | C43x |  |  |  |  |  |  |
|  |  |  |  |  |  |  |  |  |
| Lung and bronchus | 162.x | C34x |  |  |  |  |  |  |
| All cancer cases were identified using the cancer register ICD-19 and ICD-10 diagnostic codes (data-fields 40013 and 40006) | | | | | | | | |
|  |  |  |  |  |  |  |  |  |
